# Supplementary material for: The Evaluation of Roasted Lentils (L. culinaris L.) Quick Meals as An Alternative to Meat Dishes
Source: Foods. 2023 Dec 27;13(1):99. doi: 10.3390/foods13010099 (PMC10778251; doi:10.3390/foods13010099)
Supplement: Supplementary file 1 [file foods-13-00099-s001.zip › foods-2764891-supplementary.pdf]

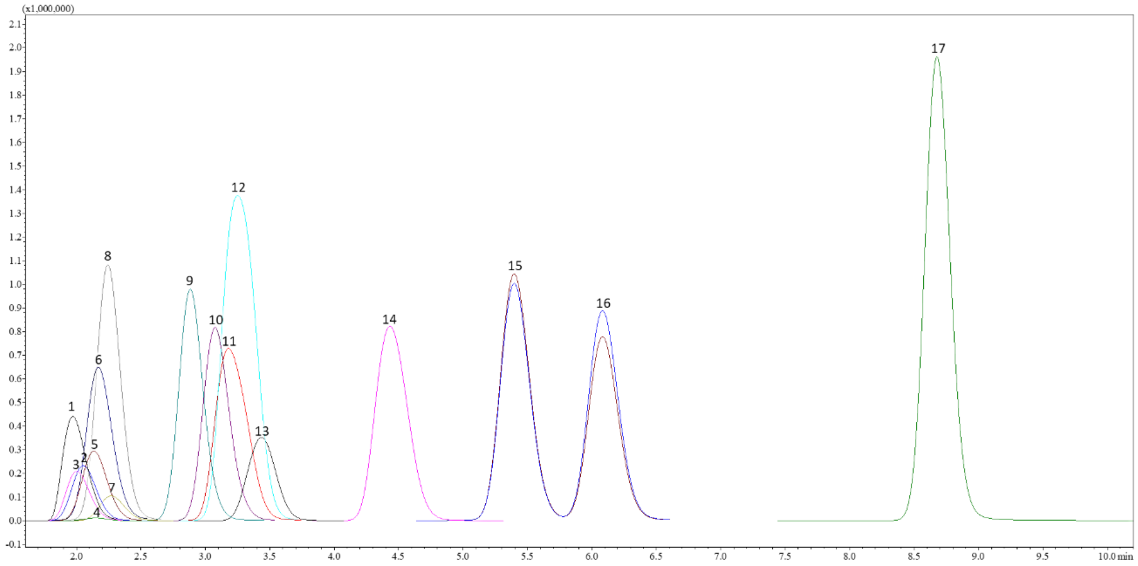

Figure S1. Selected ion chromatogram in MRM mode represents the profiles of 17 amino acids in a standard solution at the concentration of 2.5 μM L^{-1}. ***Note:*** 1—Cystine (Cys); 2—Serine (Ser); 3—Aspartic acid (Asp); 4—Glycine (Gly); 5—Threonine (Thr); 6—Glutamic acid (Glu); 7—Alanine (Ala); 8—Proline (Pro); 9—Histidine (His); 10—Lysine (Lys); 11—Valine (Val); 12—Arginine (Arg); 13—Methionine (Met); 14—Tyrosine (Tyr); 15—Isoleucine (Ile); 16—Leucine (Leu); 17—Phenylalanine (Phe).

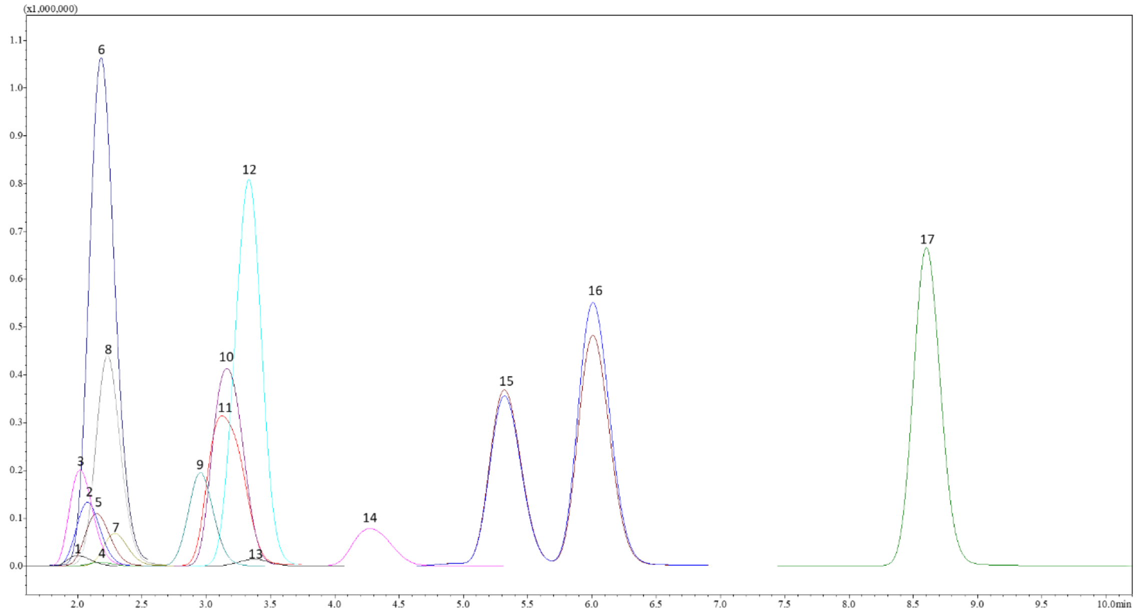

Figure S2. Selected ion chromatogram in MRM mode represents the profiles of 17 amino acids in a prepared sample of Roasted lentils. ***Note:*** 1—Cystine (Cys); 2—Serine (Ser); 3—Aspartic acid (Asp); 4—Glycine (Gly); 5—Threonine (Thr); 6—Glutamic acid (Glu); 7—Alanine (Ala); 8—Proline (Pro); 9—Histidine (His); 10—Lysine (Lys); 11—Valine (Val); 12—Arginine (Arg); 13—Methionine (Met); 14—Tyrosine (Tyr); 15—Isoleucine (Ile); 16—Leucine (Leu); 17—Phenylalanine (Phe).

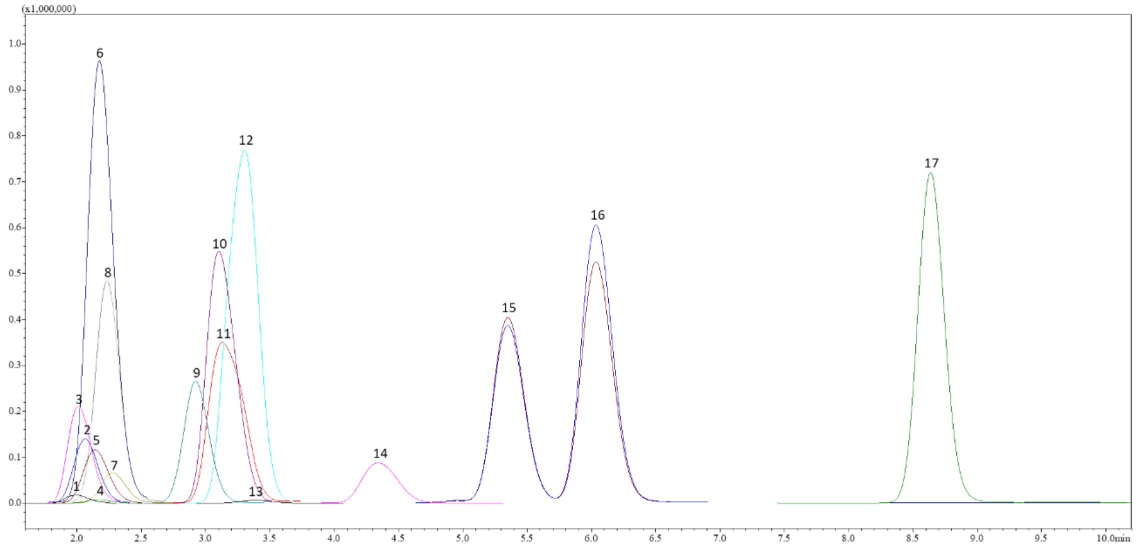

Figure S3. Selected ion chromatogram in MRM mode represents the profiles of 17 amino acids in a prepared sample of Tomatoes soup with roasted lentils. ***Note:*** 1—Cystine (Cys); 2—Serine (Ser); 3—Aspartic acid (Asp); 4—Glycine (Gly); 5—Threonine (Thr); 6—Glutamic acid (Glu); 7—Alanine (Ala); 8—Proline (Pro); 9—Histidine (His); 10—Lysine (Lys); 11—Valine (Val); 12—Arginine (Arg); 13—Methionine (Met); 14—Tyrosine (Tyr); 15—Isoleucine (Ile); 16—Leucine (Leu); 17—Phenylalanine (Phe).

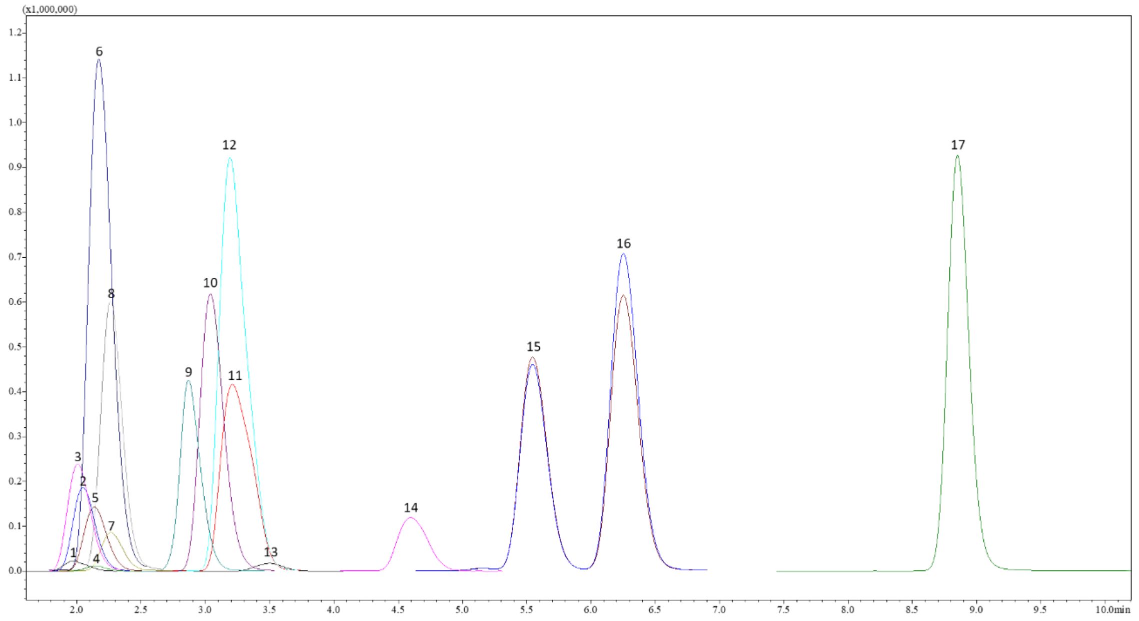

Figure S4. Selected ion chromatogram in MRM mode represents the profiles of 17 amino acids in a prepared sample of Roasted lentils with Bolognese sauce. ***Note:*** 1—Cystine (Cys); 2—Serine (Ser); 3—Aspartic acid (Asp); 4—Glycine (Gly); 5—Threonine (Thr); 6—Glutamic acid (Glu); 7—Alanine (Ala); 8—Proline (Pro); 9—Histidine (His); 10—Lysine (Lys); 11—Valine (Val); 12—Arginine (Arg); 13—Methionine (Met); 14—Tyrosine (Tyr); 15—Isoleucine (Ile); 16—Leucine (Leu); 17—Phenylalanine (Phe).
